# Biomechanical assessment of children requiring tibialis anterior surgical tendon transfer for residual congenital talipes equinovarus

**DOI:** 10.1186/1757-1146-5-S1-O31

**Published:** 2012-04-10

**Authors:** Kelly Gray, Paul Gibbons, David Little, Joshua Burns

**Affiliations:** 1 Department of Orthopaedic Surgery, The Children’s Hospital at Westmead, Sydney, NSW, 2145, Australia; 2 The University of Sydney, NSW, Australia

## Introduction

Congenital talipes equinovarus (CTEV) is a deformity in which the foot is in structural equinus, varus, adductus and cavus and occurs in approximately 1 per 1000 births [1]. Despite good initial correction with the Ponseti technique, a tibialis anterior tendon transfer (TATT) is required in 20-25% of cases to correct residual dynamic supination observed during gait. Currently, no reliable or valid biomechanical measures exist to assess the need for, or effectiveness of, surgery.

## Materials and method

Between August 2009 to April 2011, 20 children (average age 53 months ± 10 months) with CTEV were assessed prior to a TATT. Assessment included range of movement (Dimeglio Scale), foot alignment in standing (Foot Posture Index), strength (hand held dynamometry), gait (pedobarography) and function (Clubfoot Disease Specific Index). These results were compared to 12 children (average age 48 months ± 12 months) with CTEV who did not require a TATT (controls).

## Results

Range of movement and function was significantly less in the TATT group (p=0.001, p=0.006). The TATT group displayed significantly greater supination on the Foot Posture Index (p= 0.032) and significantly less eversion strength compared to the non-surgical group (p<0.001). During gait, feet of the TATT group had less contact area with ground (p=0.044), but increased contact time, particularly in the hindfoot (p=0.001), lateral midfoot (p=0.004) and lateral forefoot (p=0.034). Pressure-time integral was significantly higher in TATT group for medial and lateral hindfoot (p=0.027; p=0.010) and lateral midfoot (p=0.034).

## Conclusions

Children with CTEV who require a TATT display objective measureable differences compared to a non-surgical CTEV group. These measures may be useful in identifying which children require a TATT in the future.
